# Biochemical Properties and Possible Roles of Ectophosphatase Activities in Fungi

**DOI:** 10.3390/ijms15022289

**Published:** 2014-02-06

**Authors:** Anita Leocadio Freitas-Mesquita, José Roberto Meyer-Fernandes

**Affiliations:** 1 Institute of Medical Biochemistry, Federal University of Rio de Janeiro (UFRJ), CCS, Bloco H, University City, Fundão Island, Rio de Janeiro, RJ 21941-590, Brazil; 2 Institute of National Science and Technology of Structural Biology and Bioimage (INCTBEB), CCS, Bloco H, University City, Fundão Island, Rio de Janeiro, RJ 21941-590, Brazil

**Keywords:** ectophosphatase, host-pathogen interaction, fungal adhesion, fungal infection

## Abstract

Ectophosphatases are surface membrane-bound proteins whose active sites face the extracellular medium. These enzymes have been reported in several microorganisms including a large number of medically relevant fungal species. An effective technique for identifying ectophosphatases is performing phosphatase activity assays using living intact cells. Biochemical characterization of these activities has shown their differential modulation by classical phosphatase inhibitors, divalent metals and pH range. The physiological roles of ectophosphatases are not well established; however, it has been suggested that these enzymes play important roles in nutrition, proliferation, differentiation, adhesion, virulence and infection. Adhesion to host cells is the first step in establishing a fungal infection and ectophosphatases may be one of the first parasite proteins that come into contact with the host cells. Several results indicate that ectophosphatase activities increase the capacity of fungi to adhere to the host cells. In this context, the present review provides an overview of recent discoveries related to the occurrence and possible roles of ectophosphatase activities in fungal cells.

## 1. Introduction

Protein phosphorylation is undoubtedly the most common and also the best-studied post-translational modification. Indeed, most proteins in the cell can be regulated, directly or indirectly, through this mechanism. The phosphorylation status of any given protein is controlled by both protein kinases and phosphatases [1].

Protein phosphatases remove phosphate groups from various phosphorylated amino acids. The most common phosphorylation sites in eukaryotic cells are detected on serine, threonine and tyrosine residues [2]. Phosphorylation and dephosphorylation of these residues can trigger conformational changes in the protein that alter its properties [3,4].

It is noteworthy that most of the knowledge related to the nature and function of phosphatases and kinases is due to genetic analysis of these enzymes in fungi. Thus, phosphorylation and dephosphorylation of proteins is related to several fundamental biological processes in fungi, such as the cell cycle, transcription and cell differentiation [5,6]. Furthermore, these enzymes are involved in other cellular processes, such as cell wall synthesis [7], formation of hyphae [8] and maintenance of cellular integrity in stress situations [9–11].

## 2. Occurrence of Ectophosphatase Activities in Fungi Cells

Protein phosphatases may exist in soluble [4,12] or secreted forms [13–20], or even remain attached to the outer surface of the inner membrane [21–23] or the cell wall [24,25]. In this context, the ectophosphatases are surface membrane-bound proteins whose active site faces the extracellular medium [26–28]. It is noteworthy that some authors have designated secreted phosphatase activities as ectophosphatases because both activities are involved with extracellular metabolism.

Ectophosphatase activities have been reported in several microorganisms, including protozoa such as *Leishmania* [23,29], *Trypanosoma* [21,22,30], and bacteria, such as *Mycobacterium bovis* [31].

In fungi, ectophosphatases have been described in a large number of species, including *Saccharomyces cerevisiae* [25], *Sporothrix schenckii* [32], *Paracocciodiodes brasiliensis* [24], *Candida parapsilosis* [33,34], *Kluyveromyces marxianus* [35], *Aspergillus fumigatus* [36], *Fosencaea pedrosoi* [37,38], *Cryptococcus neoformans* [39], *Pseudallescheria boydii* [40], *Candida albicans* [41], *Rhinocladiella aquaspersa* [42] and *Metarhizium anisopliae* [43]. It is noteworthy that this review focuses on medically relevant fungal species.

### 2.1. Ectophosphatases

An effective technique for identifying ectophosphatases is performing phosphatase activity assays using intact cells. The artificial substrate *p*-nitrophenyl phosphate (*p*-NPP) is commonly used. The *p*-NPP hydrolysis generates inorganic phosphate (P_i_) and *p*-nitrophenol (*p*-NP), which turns yellow under alkaline conditions and can be quantified spectrophotometrically. This approach was performed with *S. cerevisiae* [25], *C. parapsilosis* [33,34], *F. pedrosoi* [37,38], *C. neoformans* [39], *P. boydii* [40], *C. albicans* [41], *R. aquaspersa* [42] and *M. anisopliae* [43].

Transmission electron microscopy has been used to confirm the localization of ectophosphatase activity in *F. pedrosoi* [37,38], *C. parapsilosis* [34] and *P. boydii* [40]. Cerium phosphate deposits were observed on the cell wall of the three organisms. Furthermore, in *C. parapsilosis*, precipitates were also observed in the plasma membrane and in intracellular structures, resembling vesicles [34].

In *K. marxianus*, an acid phosphatase called Pho610 was the first example of a naturally occurring cell wall protein that has enzyme activity. Pho610 was cloned in *S. cerevisiae* and the deduced amino acid sequence exhibited hydrophobic stretches at both the *N*- and *C*-termini, which is characteristic of the precursors of glycophosphatidylinositol (GPI)-anchored proteins. The results also showed that a significant portion of the Pho610 polypeptide was covalently linked to the cell wall glucan [35]. Similarly, an acid phosphatase (PhoAp) was identified as a cell wall component of *A. fumigatus*. As observed with *K. marxianus*, this is a GPI-anchored protein. However, it was not possible to determine if the type of association between PhoAp and the cell wall glucans is covalent or not [36].

### 2.2. Secreted Phosphatases

Cell-free extracts of mycelia, conidia and yeast of *S. schenckii* were submitted to polyacrylamide gel electrophoresis that showed the presence of an acid phosphatase [32]. A secreted phosphatase has also been identified in *S. cerevisiae* in a study involving both cell-free preparations and intact cells [25]. Recently, it was observed that conidial forms of *M. anisopliae* express both ectophosphatase and secreted phosphatase activity [43]. This ectophosphatase activity has been fully characterized and comparison with a secreted phosphatase activity reported in the mycelial form of the same fungus indicates that they have different biochemical properties [43,44].

## 3. Biochemical Properties of Fungal Ectophosphatases

Ectophosphatases are hydrolases that act on ester bonds and generates P_i_ as final product. For this reason, they are classified as phosphomonoesterases (EC 3.1.3). These phosphatases can be classified according to their cellular location, comprising intracellular enzymes [4,12], secreted enzymes [14,17,18] or surface enzymes that can be associated with the plasma membrane [21–23] or cell wall [24,25]. Other important tools for the classification of phosphatases are the use of inhibitors, divalent cations and different pH ranges [45]. The various activities present in fungal membranes have shown to be differently modulated by those factors.

### 3.1. Substrate Specificity of Ectophosphatase Activities

Phosphatases that act specifically on phosphoserine and phosphothreonine are known as phospho-serine/threonine phosphatases (PPs). Their mechanism of catalysis involves a nucleophilic attack from a water molecule to the phosphorylated residue, which occurs in a single step without the transference of the phosphate to the enzyme. The activation of the water molecule depends on the presence of metallic ions [46].

In contrast, enzymes that specifically hydrolyze phosphotyrosine are known as phosphotyrosine phosphatases (PTPs). Unlike PPs, PTPs do not require metal ions for catalysis.

The mechanism of catalysis for PTPs involves the formation of a phosphorylated cysteine intermediate that occurs due to the nucleophilic attack of its thiol group [47].

A third group of phosphatases, dual-specific phosphatases, are capable of hydrolyzing phosphotyrosine as well as phosphoserine and phosphothreonine residues. However, these enzymes are commonly classified as a subfamily of PTPs because of the similarities in their active site and mechanism of catalysis [47].

In general, fungal ectophosphatase activities are acid phosphatases that present high affinity for phosphotyrosine analog substrates such as *p*-NPP [25,32–39,42,43,48]. Hydrolysis of phosphorylated amino acids was examined in the mycelial and conidial forms of *F. pedrosoi* [37,38], *C. parapsilosis* [34] and *P. boydii* [40]. Phosphotyrosine was the best substrate in most cases, except for conidial forms of *F. pedrosoi*, whose ectophosphatase activity was increased three-fold when phosphothreonine was used as the substrate [38].

### 3.2. pH Influence on Ectophosphatase Activities

In yeast, acid phosphatases are localized mostly in the cell wall and the periplasmic space [49]. This is in contrast with the alkaline phosphatases, which are considered intracellular enzymes [50,51]. Accordingly, acid ectophosphatases occur much more commonly than alkaline phosphatases. Studies with *S. cerevisiae* [25], *S. schenckii* [32], *C. parapsilosis* [33,34], *K. marxianus* [35], *A. fumigatus* [36], *F. pedrosoi* [37,38], *C. neoformans* [39], *P. boydii* [40], *R. aquaspersa* [42] and *M. anisopliae* [43] have described acid phosphatases in these species.

Despite being classified as intracellular enzymes, alkaline phosphatases have also been reported as ecto-enzymes in *P. brasiliensis* [24], *C. parapsilosis* [33] and *P. boydii* [40]. In a study with *C. parapsilosis*, 24 isolates showed acid ectophosphatase activities; however, five of them also exhibited alkaline phosphatase activities. It is uncertain whether this phosphatase activity was located on the cell wall of *C. parapsilosis* or whether the enzyme seeped into a subsurface or exterior location during cell growth and maturation. However, it is possible to confirm that they are ectophosphatase activities because they have been quantified on intact cells [33].

Intact mycelial forms of *P. boydii* have been shown to express both acid and alkaline ectophosphatases. Transmission electron microscopy has confirmed that both phosphatase activities are located in the cell wall, though alkaline phosphatase activity seemed to be predominant because a large area of cerium phosphate precipitates was exhibited at pH 8 [40].

### 3.3. Modulation of Ectophosphatase Activities by Divalent Metals

It is well known that ectophosphatase activities can be positively and/or negatively modulated by cations [52]. Positive modulations were observed in *F. pedrosoi* [37,38], *C. parapsilosis* [34], *P. boydii* [40] and *R. aquaspersa* [42].

Conidial forms of *F. pedrosoi* express a Fe^3+^-activated ectophosphatase activity. However, the ectophosphatase activity from conidial forms that have grown in the absence of P_i_ in culture medium is insensitive to any metals. These results indicate that different ectophosphatases are expressed in response to exogenous P_i_ content [38].

The alkaline ectophosphatase activity of *P. boydii* has been shown to be stimulated by the following three cations: Mg^2+^, Mn^2+^ and Zn^2+^ [40]. In *R. aquaspersa*, Co^2+^ was able to promote an increase in ectophosphatase activity in a dose-dependent manner [42]. A Co^2+^-activated ectophosphatase activity was also reported in *T. brucei* [53]. Furthermore, cobalt is known to be capable of inducing the expression of alkaline phosphatase in bacteria [54].

Ectophosphatase activity in *C. parapsilosis* has been stimulated by Cu^2+^ and inhibited by Zn^2+^, both acting in a dose-dependent manner [34]. Zn^2+^ is also an ectophosphatase inhibitor in *A. fumigatus* [36], *C. neoformans* [39] and *M. anisopliae* [43]. Ectophosphatase activity in the intact mycelial forms of *P. boydii* and the conidial forms of *M. anisopliae* has been inhibited by Cd^2+^ and Cu^2+^ [40,43]. The inhibitory effects of Zn^2+^, Cd^2+^ and Cu^2+^ could be explained by the oxidizing effects that these metals can cause for the enzyme, as has been shown for other ectophosphatase activities [34,43,55].

### 3.4. Sensibility of Ectophosphatase Activities to Classical Phosphatase Inhibitors

Sodium orthovanadate is a phosphate analog. It acts as an irreversible inhibitor that binds to enzymes by adopting a trigonal bi-pyramidal structure observed in transition state during phosphoryl transfer [56]. This inhibitor can affect different biological processes [57]; however, its major biological activity in living cells is on the cell surface, as the oxidation-reduction reactions that take place in the cytoplasm diminish its inhibitory effect [56,58].

Sodium orthovanadate was able to inhibit ectophosphatase activities in a dose-dependent manner in *F. pedrosoi* [37], *C. neoformans* [39], *C. parapsilosis* [34], *P. boydii* [40] and *R. aquaspersa* [42]. Ectophosphatase activity from *A. fumigatus* was completely abolished by treatment with 10 mM of sodium orthovanadate [36]. Moreover, treatment with 1 mM of sodium orthovanadate inhibited 90% and 85% of ectophosphatase activities of *C. albicans* [41] and *M. anisopliae* [43], respectively. In *F. pedrosoi* [37,38], *C. neoformans* [39], *C. parapsilosis* [34], *P. boydii* [40], *C. albicans* [41] and *R. aquaspersa* [42], sodium orthovanadate inhibition of ectophosphatase activities was shown to be irreversible because removal of the inhibitor does not rescue the basal activity.

Ammonium molybdate and sodium fluoride are classical acid phosphatase inhibitors [59] that were also tested on several fungal ectophosphatase activities. In *F. pedrosoi* [37], *C. neoformans* [39], *C. parapsilosis* [34], *P. boydii* [40] and *R. aquaspersa* [42], both ammonium molybdate and sodium fluoride promote dose-dependent inhibition of ectophosphatase activities. In *A. fumigatus*, 10 mM ammonium molybdate and sodium fluoride were able to inhibit 85% and 60% of ectophosphatase activity, respectively [36]. Interestingly, *C. albicans* ectophosphatase activity was also inhibited 85% and 60% by, ammonium molybdate and sodium fluoride, respectively [41]. However, in this case, the concentration of the inhibitors was 1 mM, 10 times lower than the concentration used in *A. fumigatus* [36]. Only the inhibition of ectophosphatase activity by ammonium molybdate in *P. boydii* was shown to be irreversible [40].

Tartrate is a classical inhibitor for secreted phosphatases that can be used to confirm localization of the observed activity [20]. Indeed, tartrate was able to inhibit the secreted phosphatases present in *S. schenckii* [32]; however, this inhibitor was unable to modulate several ectophosphatase activities, as observed in *A. fumigatus* [36], *F. pedrosoi* [37,38]*, C. neoformans* [39] and *R. aquaspersa* [42].

Levamisole is another classical alkaline phosphatase inhibitor [60] and 1 mM of this compound was able to inhibit approximately 40% of the alkaline ectophosphatase activity in *P.boydii* [40]. In contrast, acid ectophosphatase activities seemed to be insensitive to levamisole, as observed in *F. pedrosoi* [37,38], *C. neoformans* [39], *C. parapsilosis* [34], *R. aquaspersa* [42] and *M. anisopliae* [43].

P_i_ is considered a highly specific inhibitor of phosphatase activities because it is the natural product of reactions catalyzed by phosphatases [37,38]. Inhibition by P_i_ in phosphatase reaction medium was verified in *S. cerevisiae* [25], *S. schenckii* [32], *F. pedrosoi* [37,38], *C. neoformans* [39], *C. parapsilosis* [34], *P. boydii* [40], *C. albicans* [41], *R. aquaspersa* [42] and *M. anisopliae* [43]. The P_i_ content in the culture medium is also involved in the modulation of several ectophosphatase activities, as will be discussed in the following section.

### 3.5. Modulation of Ectophosphatase Activities by Exogenous P_i_ Content

The induction of phosphatase activity in response to P_i_ starvation is an important phenomenon because these enzymes are able to hydrolyze phosphorylated substrates and supply a P_i_ source during nutrient shortages [61].

The existence of phosphatase activities regulated by phosphate in the culture medium has been reported in a variety of microorganisms, including prokaryotes species [62,63], parasitic protozoa [64,65] and fungal species including *Neurospora crassa* [66], *Aspergillus niger* [67], *Yarrowia lipolytica* [68], *Pichia pastoria* [69], *Penicillium chrysogenum* [70,71] and *S. cerevisiae* [72].

In *S. cerevisiae*, the phosphate signal transduction pathway, known as the PHO pathway, regulates the expression of several genes involved in the availability and absorption of P_i_ from extracellular sources [73]. Consequently, the transcription of genes encoding acid and alkaline phosphatases and the P_i_ transporters is coordinately repressed and de-repressed depending on the P_i_ concentration in the culture medium [74].

Most of the phosphatases synthesized under P_i_-limiting conditions are either located extracellularly or are associated with the plasma membrane or cell wall [75]. The PhoAp activity of *A. fumigatus* was significantly repressed by the presence of increasing P_i_ concentrations in culture medium [36]. Phosphate is essential for the growth of all fungi [76], and *A. fumigatus* was unable to grow at a P_i_ concentration lower than 10 μM. Because of the regulation by phosphates, it is possible to presume that PhoAp is part of an enzymatic arsenal that allows *A. fumigatus* to utilize P_i_ from its environment [36].

In *F. pedrosoi*, cultivation of conidia in the absence of exogenous P_i_ resulted in a generation of fungal cells expressing an ectophosphatase activity 130-fold higher than that expressed by fungi cultivated in the presence of P_i_. The depletion of P_i_ from the culture medium apparently induced the expression of a different ectophosphatase, as suggested by: the differences in the affinity for the artificial substrate *p*-NPP; by the fact that both activities are differently modulated by metals; and by the differential sensitivity to classical phosphatase inhibitors [37,38]. Recently, similar results were obtained with *R. aquaspersa*. Removal of P_i_ from the culture medium of conidial forms resulted in a 121-fold increase in their ectophosphatase activities, when compared with the activities of conidia grown in the presence of P_i_ [42].

## 4. Possible Roles of Fungal Ectophosphatase Activities

Despite having been described in several microorganisms, the physiological roles of ectophosphatases in these cells are not well established. It has been suggested that these enzymes play important roles in nutrition, proliferation, differentiation, adhesion, virulence and infection [45].

The ectophosphatases may provide fungal cells with a source of orthophosphate by hydrolyzing organic phosphates [77]. In other microorganism, it was observed that the process of adhesion to mammalian cells requires energy [78]. Since P_i_ is the only phosphorous source readily available to be taken up and metabolized by microorganisms, ectophosphatase activity could play a pivotal role in the release of P_i_ during the first stages of infection, a moment that is not easy to exactly define and when the fungus probably is still not fully metabolically active [43,79].

Moreover, ectophosphatases may protect the cells from acidic conditions by buffering the periplasmic space with phosphates released from polyphosphates [80]. There is also evidence that ectophosphatases are involved in cell wall synthesis, remodeling and degradation [81].

In this section, we will discuss some recent reports that describe possible roles for ectophosphatase activity in fungal cells.

### 4.1. Differential Expression of Fungal Ectophosphatase Activities

The differentiation process is a fundamental event during the fungal life cycle. Interestingly, distinct levels of ectophosphatase activities can be observed in the different fungal morphological forms. Sclerotic bodies, conidial and mycelial forms of *F. pedrosoi* and *R. aquaspersa*, two etiological agents in chromoblastomycosis (CBM), have been shown to be able to hydrolyze the artificial substrate *p*-NPP. In *R. aquaspersa*, the conidial form presented higher levels of ectophosphatase activity when compared with the other two forms. In contrast, *F. pedrosoi* sclerotic bodies exhibited the highest ectophosphatase activities. As conidial forms are the putative infectious propagules in CBM and sclerotic bodies are the prevalent fungal forms observed in the tissue lesions, it is probable that ectophosphatase activity is associated with parasitism [37,42].

The *in vitro* differentiation of *F. pedrosoi* mycelia to sclerotic forms, induced by platelet-activating factor, concomitantly stimulates the ectophosphatase activity [48]. Furthermore, a strain recently isolated from a human case of CBM also demonstrated increased ectophosphatase activity suggesting that the expression of ectophosphatases in *F. pedrosoi* may be stimulated by interaction with the host [37].

The expression and/or activity of ectophosphatases in fungal species may vary in the different isolates. Different cryptococcal isolates have presented ectophosphatase activity, indicating that this enzyme is commonly expressed in *C. neoformans* strains; however, considerable variation in expression levels were observed between the different isolates [39].

*Candida* species usually exist as commensals in the oral, gastrointestinal and genital mucosa of healthy individuals. However, these fungi are also opportunistic pathogens that may cause a wide variety of superficial and systemic infections if the host immune system is compromised [82]. Interestingly, a strong association has been identified between the ectophosphatase activities of *C. albicans* and HIV infection [41].

Superficial isolates from *C. parapsilosis* exhibited significantly greater ectophosphatase activity when compared with their systemic counterparts. Moreover, the adhesion to human buccal epithelial cells was also increased in assays performed with superficial isolates of *C. parapsilosis*. The determination that the survival of the surface dwellers is critically dependent on their ability to attach suggests that ectophosphatases are involved in metabolic events on the interface between the host and the yeast and thereby play a role in candidal adhesion to host surfaces [33].

### 4.2. Involvement of Fungal Ectophosphatase Activities in Host-Parasite Interactions

To establish an infection, pathogens have to evade the immune system, survive and divide in the host environment and spread to new tissues [83,84]. Ecto-enzymes are located in the plasma membrane with their active sites facing the external environment, and could be one of the parasite proteins that initially come into contact with the host cells in the invasion process [27]. This peculiar location suggests that these enzymes may be involved in virulence and infection [27,45].

The irreversible profile of enzyme inhibition produced by sodium orthovanadate in *F. pedrosoi* [38], *C. neoformans* [39], *C. parapsilosis* [34], *C. albicans* [41], *R. aquaspersa* [42] and *M. anisopliae* [43] has allowed the comparison of the ability of these fungi to attach to host cells when ectophosphatase activity was fully functional and when surface enzyme activity was inhibited by pretreatment of fungal cells with this inhibitor. This pretreatment of fungal cells with orthovanadate inhibited adhesion to epithelial cells in *F. pedrosoi* and *R. aquaspersa*, two etiological agents in CBM [38,42], *C. neoformans*, the causative agent in cryptococcosis [39], and *C. parapsilosis* and *C. albicans*, two important agents in human candidiasis [33,41]. In *F. pedrosoi*, pretreatment with orthovanadate also decreased the association of the conidial forms to fibroblasts [38].

*M. anisopliae* is an entomopathogenic fungus with the ability to infect a broad range of arthropods, from ticks and agricultural insect pests to vectors of human diseases. Corroborating the results observed in human pathogens, pretreatment with phosphatase inhibitors (orthovanadate and molybdate) led to a decrease in the adhesion of *M. anisopliae* to the model host *Dysdercus peruvianus* fly wings. Moreover, bioassays have revealed an increase in the survival of *D. peruvianus* infected by *M. anisopliae* when conidial forms were pre-incubated with different phosphatase inhibitors, suggesting that ectophosphatase activity plays an important role in fungal virulence [43]. Conidial forms of *F. pedrosoi* and *R. aquaspersa* cultured in P_i_-depleted medium presented higher levels of ectophosphatase activity and also showed greater adherence to cultured fibroblasts or epithelial cells than the cells grown in complete medium [38,42].

All the reported adhesion assays have shown that cells expressing high ectophosphatase activities were significantly more capable of adhering to host cells. These mechanisms seem to be conserved not only in fungi but also in protozoa. Recently, the involvement of ectophosphatases in host-pathogen interaction was described in *Trypanosoma rangeli* [30] and *Strigomonas culicis* [85].

**Figure 1.**
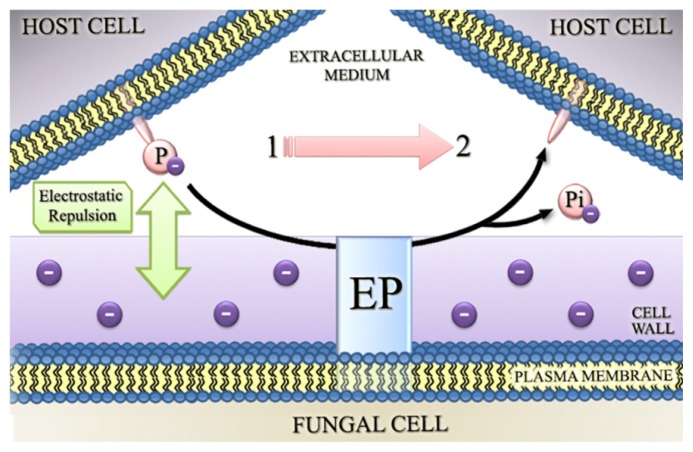
The removal of phosphate groups from surface proteins in host cells could result in attenuation of the electrostatic repulsion between fungal and host cells. The fungal cell surface is electronegative and the presence of phosphate residues on the surface of the host cell increases the electrostatic repulsion between them. Ectophosphatases may contribute to the success of fungal infection by removing phosphate residues of the host cell plasma membrane and thereby attenuating this electrostatic repulsion. Abbreviations: EP, ectophosphatase; P, phosphate residue; P_i_, inorganic phosphate; 1, before ectophosphatase activity; and 2, after ectophosphatase activity.

**Figure 2.**
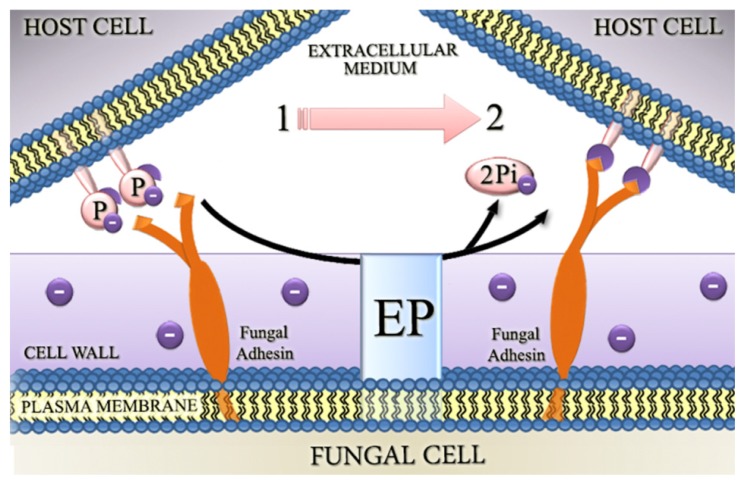
The removal of inorganic phosphate could expose additional sites for the interaction of infectious agents with the host surface. Phosphorylated proteins may contain binding sites for fungal adhesins. Dephosphorylation promoted by ectophosphatase activity could therefore expose these sites, thus contributing for fungal adhesion and infection. Abbreviations: EP, ectophosphatase; P, phosphate residue; P_i_, inorganic phosphate; 1, before ectophosphatase activity; and 2, after ectophosphatase activity.

**Figure 3.**
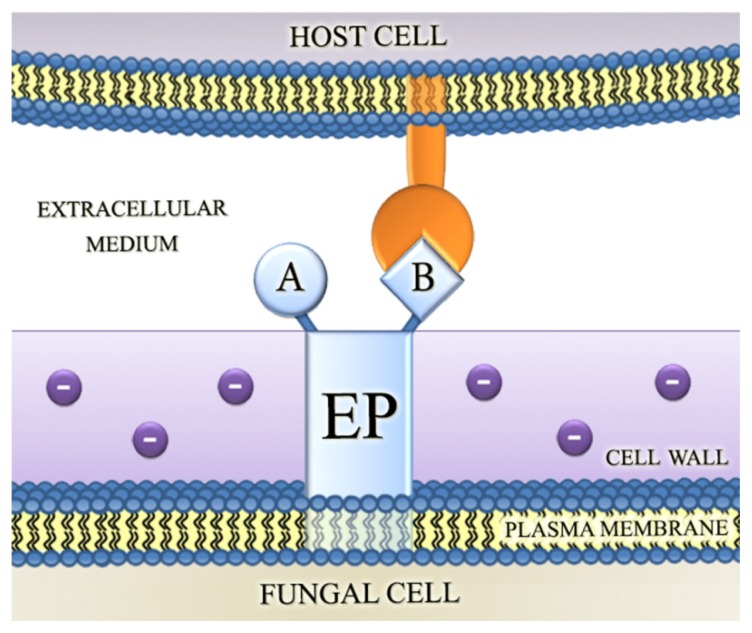
Ectophosphatase may contain adhesive domains that could directly promote the attachment of fungal cells to their hosts. In addition to the catalytic domain, ectophosphatase may also exhibit an adhesion domain, thus functioning similarly to the well-characterized microbial adhesins. Abbreviations: EP, ectophosphatase; A, catalytic domain; and B, adhesion domain.

To explain the involvement of ectophosphatase in host-parasite interaction, the following possible roles for this enzyme were proposed: (1) the removal of phosphate groups from surface proteins in host cells could result in conformational transitions and in an attenuated electrostatic repulsion between fungal and host cells (Figure 1); (2) the removal of P_i_ could expose additional sites for the interaction of infectious agents with the host surface (Figure 2); and (3) ectophosphatases may contain adhesive domains that could directly promote the attachment of fungal cells to their hosts, therefore functioning similarly to the well-characterized microbial adhesins (Figure 3) [34,38,39,41–43].

## 5. Conclusion

Throughout this review, several studies were reported describing the occurrence of ectophosphatase activities in the plasma membranes of fungal cells. The biochemical characterization of these activities has shown their differential modulation by classical phosphatase inhibitors, divalent metals and pH. Furthermore, several results indicated that ectophosphatase activity increases the capacity of fungi adherence on host cells. Clearly, more studies are needed to determine the extent of ectophosphatase participation in fungal physiology, as well as in host-pathogen interactions. The possible correlations between the expression of these enzymes and the clinical manifestation of diseases make them a potential therapeutic target.
